# Surveillance of endemic human coronaviruses (HCoV-NL63, OC43 and 229E) associated with childhood pneumonia in Kilifi, Kenya

**DOI:** 10.12688/wellcomeopenres.16037.2

**Published:** 2020-09-22

**Authors:** Grieven P. Otieno, Nickson Murunga, Charles N. Agoti, Katherine E. Gallagher, Juliet O. Awori, D. James Nokes

**Affiliations:** 1 Epidemiology and Demography Department, Kenya Medical Research Institute-Wellcome Trust Research Programme, Kilifi, Kenya; 2 London School of Hygiene and Tropical Medicine,, London, UK; 3 School of Life Sciences and Zeeman Institute for Systems Biology and Infectious Disease Epidemiology Research (SBIDER), University of Warwick, Coventry, UK

**Keywords:** Human coronavirus, NL63, OC43, 229E

## Abstract

**Introduction: **Human coronaviruses (HCoVs) circulate endemically in human populations, often with seasonal variation. We describe the long-term patterns of paediatric disease associated with three of these viruses, HCoV-NL63, OC43 and 229E, in coastal Kenya.

**Methods:** Continuous surveillance of pneumonia admissions was conducted at the Kilifi county hospital (KCH) located in the northern coastal region of Kenya. Children aged <5 years admitted to KCH with clinically defined syndromic severe or very severe pneumonia were recruited. Respiratory samples were taken and tested for 15 virus targets, using real-time polymerase chain reaction. Unadjusted odds ratios were used to estimate the association between demographic and clinical characteristics and HCoV positivity.

**Results:** From 2007 to 2019, we observed 11,445 pneumonia admissions, of which 314 (3.9%) tested positive for at least one of the HCoV types surveyed in the study. There were 129 (41.1%) OC43, 99 (31.5%) 229E, 74 (23.6%) NL63 positive cases and 12 (3.8%) cases of HCoV to HCoV coinfection.  Among HCoV positive cases, 47% (n=147) were coinfected with other respiratory virus pathogens. The majority of HCoV cases were among children aged <1 year (66%, n=208), though there was was no change in the proportion infected by age. HCoV-OC43 was predominant of the three HCoV types throughout the surveillance period. Evidence for seasonality was not identified.

**Conclusions:** Overall, 4% of paediatric pneumonia admissions were associated with three endemic HCoVs, with a high proportion of cases co-occurring with another respiratory virus, no clear seasonal pattern, and with the age-distribution of cases following that of pneumonia admissions (i.e. highest in infants). These observations suggest, at most, a small severe disease contribution of endemic HCoVs in this tropical setting and offer insight into their potential future burden and epidemiological characteristics.

## Introduction

To date, seven human coronaviruses (HCoVs) have been identified, of which four (NL63, HKU1, OC43 and 229E) are known to be endemic among humans ^1– 4^. Endemic HCoV types are detected in a small but non-negligible proportion of respiratory tract infections; mild cases occur across a wide age-range and severe disease is predominant in young children and the elderly ^5– 8^. A further three HCoVs have emerged in recent years and caused epidemics: SARS-CoV the agent of severe acute respiratory syndrome in China ^9^, MERS-CoV the cause of Middle East respiratory syndrome in the Middle-East ^10^ and most recently SARS-CoV-2, the aetiological agent of the current pandemic of coronavirus disease 2019 (COVID-19) ^11^. To date, there are limited preventive options against HCoV infections and no effective anti-viral treatment ^12^. Understanding the epidemiology of HCoVs can play a critical role in prediction, prevention and control of HCoV infection. In addition, data on endemic HCoVs may inform expectations for SARS-CoV-2 if it becomes endemic. Our study aims to describe the circulation patterns of three endemic HCoVs (OC43, 229E, and NL63) over time using data from a long-term surveillance programme in a rural coastal setting in Kenya, 3° south of the equator.

## Methods

### Study setting

A prospective study was established in 2007 for long-term continuous respiratory virus surveillance among pneumonia admissions to Kilifi County Hospital (KCH) ^6^ in order to develop improved epidemiological understanding, estimate disease burden and provide suitable baseline data for future vaccine studies. KCH is the referral hospital within the Kilifi Health and Demographic Surveillance System (KHDSS), in the northern coastal region of Kenya ^13^. The location experiences two rainy seasons approximately from April to July and October to December, with median maximum temperature of 33°C (IQR: 31–36), median minimum temperature of 23°C (IQR: 22–24), and median relative humidity of 78% (IQR: 71–87) (unpublished weather station data). Children aged 1 day to 59 months admitted to KCH with clinical symptoms of severe or very severe pneumonia were recruited. Written informed consent was sought from parents/guardians of the children prior to sample collection. In this paper we define severe pneumonia as history of cough or difficulty breathing and chest indrawing while very severe pneumonia is defined as history of cough or difficult breathing and at least one of inability to feed, prostration, unconsciousness, or oxygen saturation of <90% by fingertip pulse oximetry. We use the term pneumonia to refer to all cases of clinically severe or very severe pneumonia ^14^. The variables extracted from the hospital surveillance database include; Demographic characteristics (sex, KHDSS residency status, age), presence/absence of clinical features (history of cough, difficulty breathing, cyanosis, nasal flaring, chest indrawing, crackles, wheeze, inability to drink, vomits everything, fever defined as axillary temperature ≥37.5° C, oxygen saturation levels, conscious level: agitated, lethargic, prostration or unconscious, pneumonia status: severe or very severe), laboratory test results, and hospitalisation outcomes (admission to the high dependency unit, discharge outcomes: alive or dead)

### Laboratory methods

Specimens collected between January 2007 and December 2019 were processed and screened for three HCoVs (OC43, NL63 and 229E) and at least 12 other respiratory viral pathogens (RSV (A and B), rhinovirus, HCoVs (NL63, OC43, 229E), influenza (A, B and C), parainfluenza virus (1–4), adenovirus, and human metapneumovirus) using real-time polymerase chain reaction (RT-PCR). Sample testing was initially performed in 2007 using the LightCycler Fast Start DNA MasterPLUS HybProbe kit (Roche) ^6^, then multiplex RT-PCR using Qiagen Quantifast multiplex RT-PCR kit (Qiagen, United Kingdom) in triplex sets on an ABI 7500 system, from January 2007 until the present day ^15, 16^; additionally, a proportion of samples were tested using a 33-pathogen multiplex quantitative PCR (FTD Resp-33, Fast Track Diagnostics, Sliema, Malta) as part of the multi-country PERCH study ^17^, between August 2011 and December 2013. A variety of collection methods was used: nasal wash (2007 to 2009), nasopharyngeal flocked swab (2010 to 2014), or combined nasopharyngeal swab and oropharyngeal swab (2015 onwards).

### Data analysis

Data analysis was done using STATA version 13.0 (Stata Corp, College Station Texas, USA). Summary statistics (counts, proportions, measures of central tendency and variation) are presented for continuous and categorical data as appropriate. We estimated unadjusted odds ratios to measure the association between demographic and clinical characteristics of the study participants and testing positive for HCoV. Three Poisson regression models, one for each HCoV type, were used to investigate the presence of seasonality. In the models a trend variable was included and residuals plotted against month. Identification of a strong pattern by visual inspection of the residual plots would suggest presence of seasonality. The chi-square test of proportional trends was used to test for a linear trend in the proportions of samples tested or not tested for HCoV over time. To check for an association between categorical variables the chi-square test of association or Fisher’s exact test was used as appropriate. The analysis code is provided as *Extended data* ^18^.

### Ethical approval

This study was approved by the Kenya Medical Research Institute Scientific Ethics Review Unit (Approval number: KEMRI/SERU/CGMR-C/027/3178).

## Results

### Characteristics of patients infected with HCoVs

During the 13 years of surveillance, there were 49,409 paediatric admissions of children aged 0–59 months at KCH. A total of 11,445 (23.2%) admissions were due to severe (n= 7808, 68%) and very severe (n=3637, 32%) pneumonia. Out of the eligible cases, 69.5% (n=7957) were tested for the three HCoVs while the remainder were not tested due to refusal of consent (13.8%), discharge (13%) or death (3.7%) prior to sample collection. Cases untested did not differ from those tested in age distribution or sex ratio, but were more likely to be very severe (40.0% versus 28.3%, Fishers exact P-value <0.001).

Of the 7957 children tested, 5312 (66.7%) were aged <1 year, 1454 (18.3%) were aged 12–23 months, 620 (7.8%) were aged 24–35 months and 571 (7.2%) were in the 36–59 months age band. The proportion of tested individuals with fever (axillary temperature ≥37.5°C), cough and difficulty breathing was 58.3%, 83.2%, 92.6%, respectively. A total of 314 (3.9%) tested positive for at least one of the three HCoV targets. Among the HCoV positives, 129 (41.1%) had OC43, 99 (31.5%) had 229E, 74 (23.6%) had NL63 and 12 (3.8%) had coinfections between the three HCoV types. Among all the samples tested, the overall prevalence of NL63 was 1% (n=80), 1.7% for OC43 (n=137) and 1.4% for 229E (n=109).

The characteristics of the patients positive for any and for each HCoV type or infection combination are described in Table 1. HCoV positive cases were predominantly children aged <1 year (66.2%) and those aged 12–23 months (18.2%). The burden of infection with at least one HCoV, among all pneumonia admissions, was highest in infants and decreased with increasing age (2.6% for those under 1 year and 0.7% for 12–23 months). The same pattern was seen for each individual HCoV type (not shown). However, the proportion of samples testing positive for HCoV (3.9%) did not vary with age group (Fisher’s exact p-value = 0.753). Among all HCoV positive participants, mean age was 11 months (median 7 months), there were fewer females than males and fewer with very severe compared to severe pneumonia. At least half of the HCoV positives presented with fever (58%) and nasal flaring (53%).

**Table 1.** Demographic and clinical characteristics of study children (aged under 5 years) admitted with severe or very severe pneumonia to Kilifi County Hospital, Kilifi, Kenya 2007–2019, by HCoV type (n=314).

Variable		NL63 (n=74)	OC43 (n=129)	229E (n=99)	HCoVs coinfections (n=12)	Total (n=314)
		n (%)	n (%)	n (%)	n (%)	n (%)
Age (months)	Median (IQR)	8 (3-20)	7 (2-15)	8 (2-19)	5.5 (2-8.5)	7 (2-16)
Age categories	0–11 months	49 (66.2)	87 (67.4)	61 (61.6)	12 (91.7)	208 (66.2)
	12–23 months	11 (14.9)	24 (18.6)	22 (22.2)	0 (0.00)	57 (18.2)
	24–35 months	11 (14.9)	8 (6.2)	9 (9.1)	1 (8.3)	29 (9.2)
	36–59 months	3 (4.0)	10 (7.8)	7 (7.1)	0 (0.00)	20 (6.4)
Sex	Female	22 (29.7)	60 (46.5)	36 (36.4)	6 (50.0)	124 (39.5)
	Male	52 (70.2)	69 (53.5)	63 (63.6)	6 (50.0)	190 (60.5)
Pneumonia status	Severe	49 (66.2)	101 (78.3)	76 (76.8)	10 (83.3)	236 (75.2)
	Very severe	25 (33.8)	28 (21.7)	23 (23.2)	2 (16.7)	78 (24.8)
Clinical presentation	Cough	55 (74.3)	112 (86.8)	85 (85.9)	12 (100.00)	264 (84.1)
	Difficulty breathing	67 (90.5)	118 (91.5)	94 (95.0)	10 (83.3)	289 (92.1)
	Fever *	43 (58.1)	75 (58.1)	54 (54.6)	10 (83.3)	182 (58.0)
	Prostratration or unconscious	17 (23.0)	18 (14.0)	14 (14.1)	1 (8.3)	50 (15.9)
	Chest Indrawing	65 (87.8)	122 (94.6)	89 (89.9)	12 (100.00)	288 (91.7)
	Wheeze	7 (9.5)	16 (12.5)	18 (18.2)	0 (0.0)	41 (13.1)
	Crackle	27 (36.5)	44 (34.1)	40 (40.4)	3 (25.0)	114 (36.3)
	Nasal flaring	40 (54.1)	66 (51.2)	54 (54.6)	6 (50.0)	166 (52.9)
	Shock *	12 (16.2)	10 (7.8)	6 (6.1)	0 (0.00)	28 (8.9)
	Hypoxemia (O _2_ <90%)	12 (16.2)	14 (10.9)	13 (13.1)	1 (8.3)	40 (12.7)
	Cyanosis	1 (1.4)	1 (0.8)	1 (1.0)	0 (0.00)	3 (1.0)
	Inability to drink/ feed	5 (6.8)	11 (8.5)	7 (7.1)	0 (0.00)	23 (7.3)
	Vomits everything	7 (9.5)	6 (4.7)	0 (0.00)	0 (0.00)	13 (4.14)
Duration of hospital stay	Mean (SD)	6.3 (6.8)	5.7 (6.1)	5.2 (5.5)	3.8 (2.3)	5.6 (6.0)
	Median (IQR)	4 (3-6)	3 (2-7)	3 (2-6)	6.5 (3-10)	4 (2-6)
Outcomes	HDU *	27 (36.5)	31 (24.0)	22 (22.2)	2 (16.7)	82 (26.1)
	Died	10 (13.5)	10 (7.8)	7 (7.1)	0 (0.00)	27 (8.6)

* Fever is defined as axillary temperature ≥37.5°C, shock is defined as capillary refill of >3 seconds, temperature gradient or weak pulse volume. HDU indicates study participants who were critically ill and were transferred from the general ward to the high dependency unit. ** The high proportions of cough, difficulty breathing, chest indrawing, hypoxemia, prostratration or unconscious and inability to drink/feed among HCoV positive cases should be interpreted with caution because these clinical signs form part of our study’s eligibility criteria.

### Clinical outcomes of HCoV-infected patients

**Figure 1.**
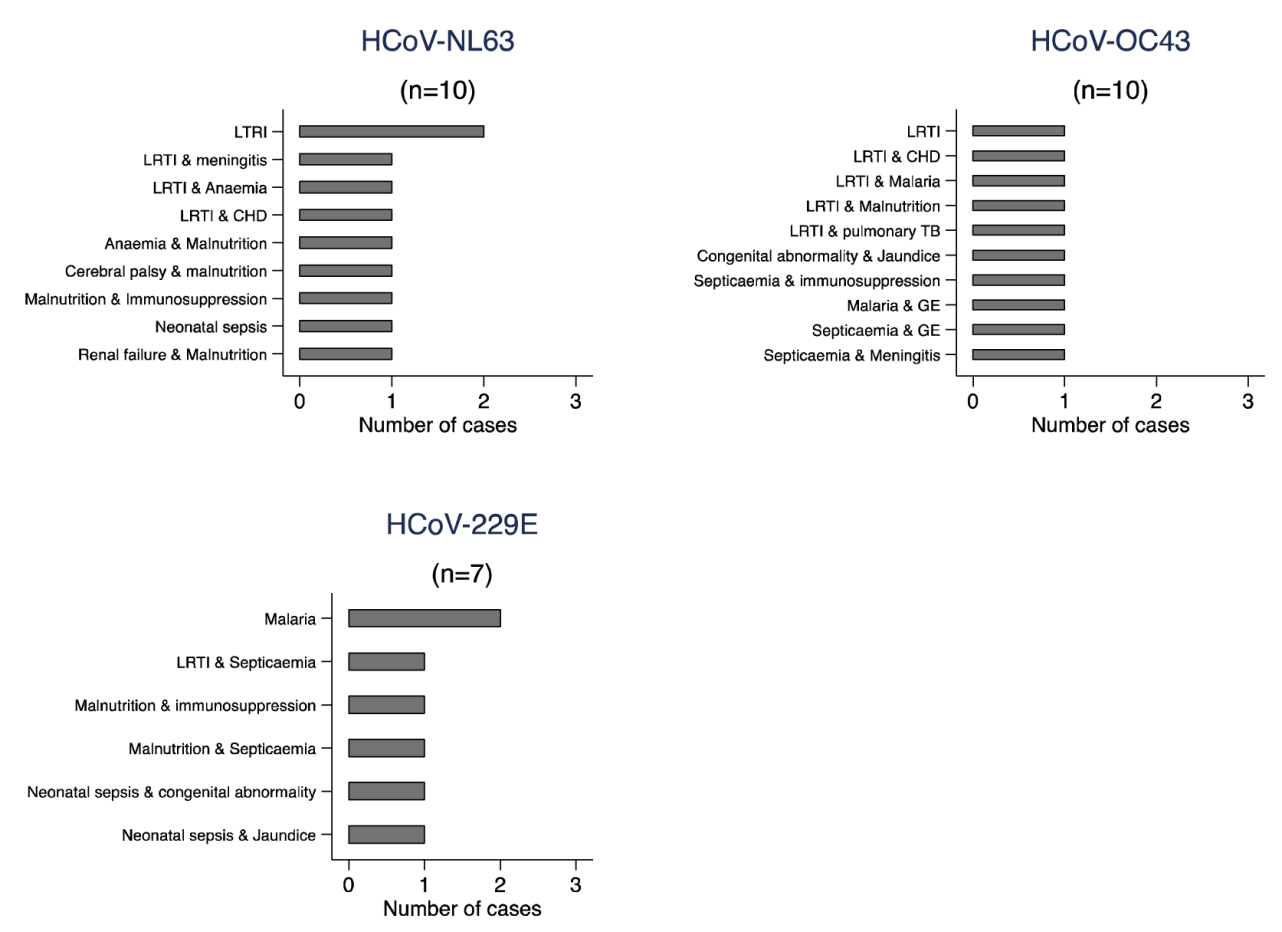
Frequency distribution of discharge diagnosis for mortality cases admitted to Kilifi County Hospital, Kilifi, Kenya 2007–2019, by HCoV type. LRTI=lower respiratory tract infection, CHD=congenital heart disease, TB=Tuberculosis, GE=Gastroenteritis.

Over a quarter of those positive for at least one of the three endemic HCoVs investigated in this study were admitted to the high dependency unit and of those positive for OC43, 13.5% (n=10) died while 7.8% (n=10) and 7.1% (n=7) died of those positive for NL63 and 229E, respectively. A large proportion of these deaths were observed among those with underlying co-morbidities ( Figure 1). None of the HCoVs were statistically significantly associated with any of the specific clinical signs or outcomes investigated (p-values>0.05) except death among NL63 cases ( Table 2); however, we had limited power to detect associations given the small number of HCoV positive cases.

**Table 2.** Unadjusted Odds Ratios (ORs) for demographic and clinical characteristics and the hospitalisation outcomes for children (under 5 years) admitted with severe or very severe pneumonia to Kilifi County Hospital, Kilifi, Kenya 2007-2019 by the 3 HCoV types.

		NL63		OC43		229E		Any HCoV	
		OR (95% CI)	p- value	OR (95% CI)	p- value	OR (95% CI)	p- value	OR (95% CI)	p- value
Age	0–11 months	Ref		Ref		Ref		Ref	
	12–23 months	0.74 (0.39-1.42)	0.369	0.92 (0.59-1.45)	0.723	1.15 (0.71-1.86)	0.569	1.00 (0.74 – 1.35)	0.994
	24–35 months	1.92 (1.02-3.61)	0.043	0.72 (0.35-1.48)	0.371	1.23 (0.63-2.39)	0.547	1.20 (0.81 – 1.79)	0.360
	36–59 months	0.51 (0.16-1.65)	0.263	0.98 (0.51-1.89)	0.949	0.93 (0.43-2.03)	0.854	0.89 (0.56 – 1.42)	0.627
Sex	Female	Ref		Ref		Ref		Ref	
	Male	1.62 (1.01-2.60)	0.046	0.81 (0.58-1.14)	0.221	1.27 (0.86-1.88)	0.233	1,13 (0.89 – 1.42)	0.302
Cough	No	Ref		Ref		Ref		Ref	
	Yes	0.64 (0.38-1.08)	0.098	1.43 (0.86-2.39)	0.168	1.37 (0.78-2.42)	0.268	1.07 (0.79 – 1.46)	0.671
Breathing difficulty	No	Ref		Ref		Ref		Ref	
	Yes	0.63 (0.31-1.27)	0.196	0.83 (0.46-1.52)	0.544	1.38 (0.61-3.17)	0.440	0.93 (0.61 – 1.41)	0.720
Fever *	No	Ref		Ref		Ref		Ref	
	Yes	1.13 (0.72-1.78)	0.587	1.04 (0.73-1.46)	0.839	0.94 (0.65-1.38)	0.766	0.99 (0.79 – 1.24)	0.908
Prostration/ unconsciousness	No	Ref		Ref		Ref		Ref	
	Yes	1.44 (0.83-2.46)	0.189	0.85 (0.52-1.39)	0.517	0.84 (0.49-1.46)	0.543	1.00 (0.74 – 1.37)	0.982
Chest indrawing	No	Ref		Ref		Ref		Ref	
	Yes	0.68 (0.33-1.37)	0.279	1.62 (0.75-3.48)	0.217	0.86 (0.44-1.65)	0.64	0.96 (0.64 – 1.44)	0.835
Inability to feed	No	Ref		Ref		Ref		Ref	
	Yes	0.54 (0.22-1.35)	0.189	0.71 (0.38-1.32)	0.283	0.62 (0.29-1.34)	0.226	0.66 (0.43 – 1.02)	0.061
Hypoxemia (O _2_<90%)	No	Ref		Ref		Ref		Ref	
	Yes	0.92 (0.51-1.67)	0.780	0.58 (0.34-0.99)	0.046	0.64 (0.34-1.14)	0.131	0.68 (0.49 – 0.96)	0.026
Pneumonia status	Severe	Ref		Ref		Ref		Ref	
	Very severe	1.22 (0.76-1.95)	0.406	0.71 (0.47-1.06)	0.094	0.71 (0.45-1.12)	0.143	0.83 (0.64 – 1.08)	0.162
Hospital stay	<= 4 days	Ref		Ref		Ref		Ref	
	> 4 days	1.06 (0.68-1.65)	0.807	0.85 (0.60-1.21)	0.373	0.72 (0.49-1.08)	0.110	0.88 (0.70 – 1.11)	0.285
Death	No	Ref		Ref		Ref		Ref	
	Yes	1.98 (1.02-3.87)	0.045	1.07 (0.56-2.05)	0.842	0.93 (0.43-2.03)	0.872	1.29 (0.86 – 1.93)	0.218

*Fever is defined as axilliary temperature ≥ 37.5° C

### Co-infection with other respiratory viruses

**Figure 2.**
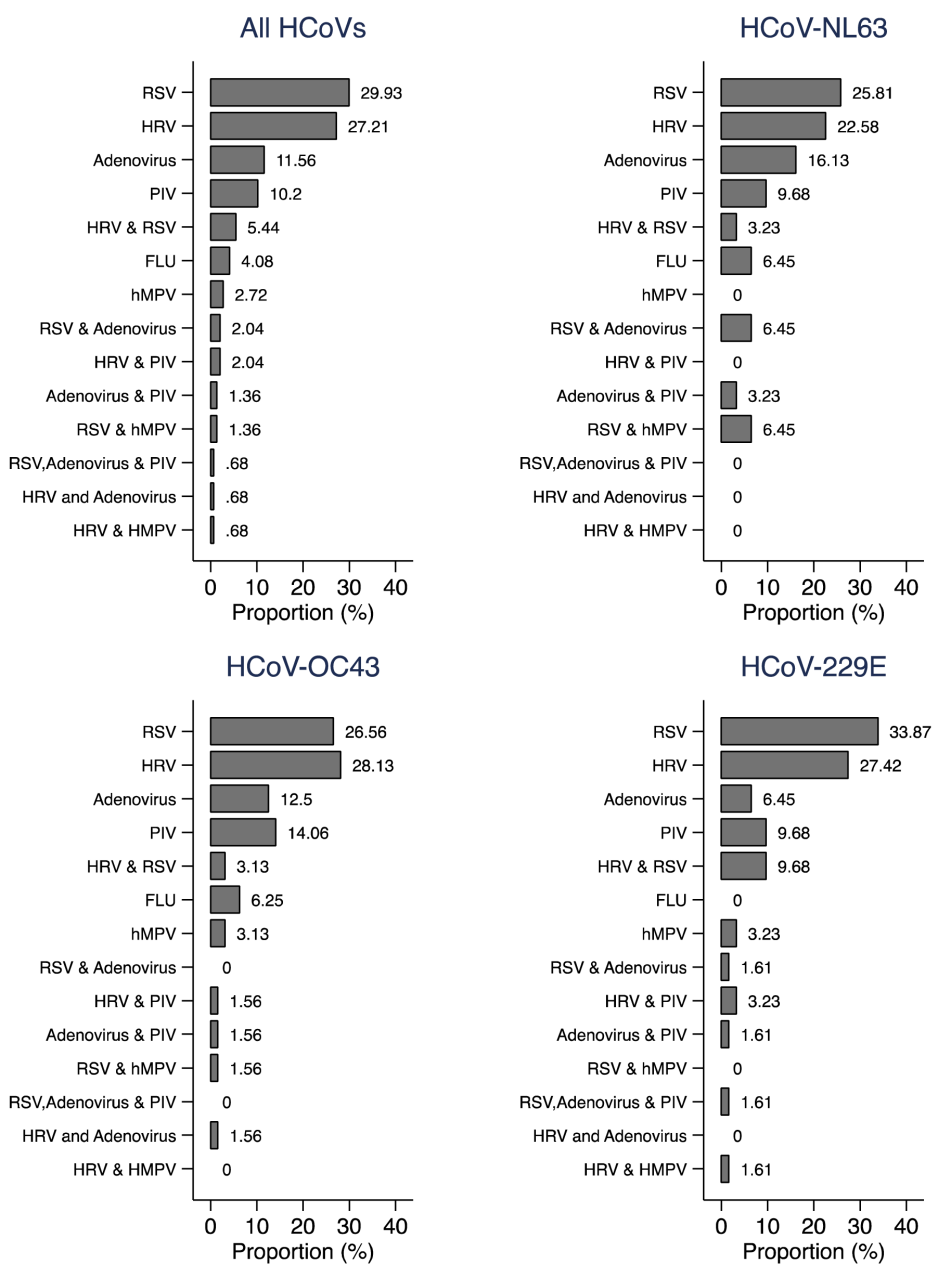
Percentage distribution of coinfections between HCoVs and other viral pathogens for pneumonia cases admitted to Kilifi County Hospital, Kilifi, Kenya 2007–2019. RSV=respiratory syncytial virus (A and B), HRV=human rhinovirus, PIV=parainfluenza, FLU=influenza (A, B and C), hMPV= human metapneumovirus.

About 47% (n=147) of the 314 HCoV cases were co-infected with other viral respiratory pathogens; respiratory syncytial virus (RSV) and human rhinovirus (HRV) jointly accounted for >50% of all HCoV coinfections with other pathogens. A similar coinfection pattern was observed for each HCoV tested ( Figure 2). Throughout the surveillance period, there were three cases (one of NL63 and two of OC43) aged <1 year that were readmitted and tested positive for the same HCoV as the first admission. The NL63 readmission occurred 10 days after discharge from the first admission while the OC43 readmissions were at 3 and 21 days after discharge from the first admission. The NL63 case had a discharge diagnosis of neonatal sepsis for the first admission and gastroenteritis plus lower respiratory tract infection (LRTI) for the readmission. One of the OC43 cases had a discharge diagnosis of LRTI for both admissions while the other had immunosuppression plus malnutrition in the first and immunosuppression plus septicaemia for the second admission.

### Temporal patterns of different HCoVs

**Figure 3.**
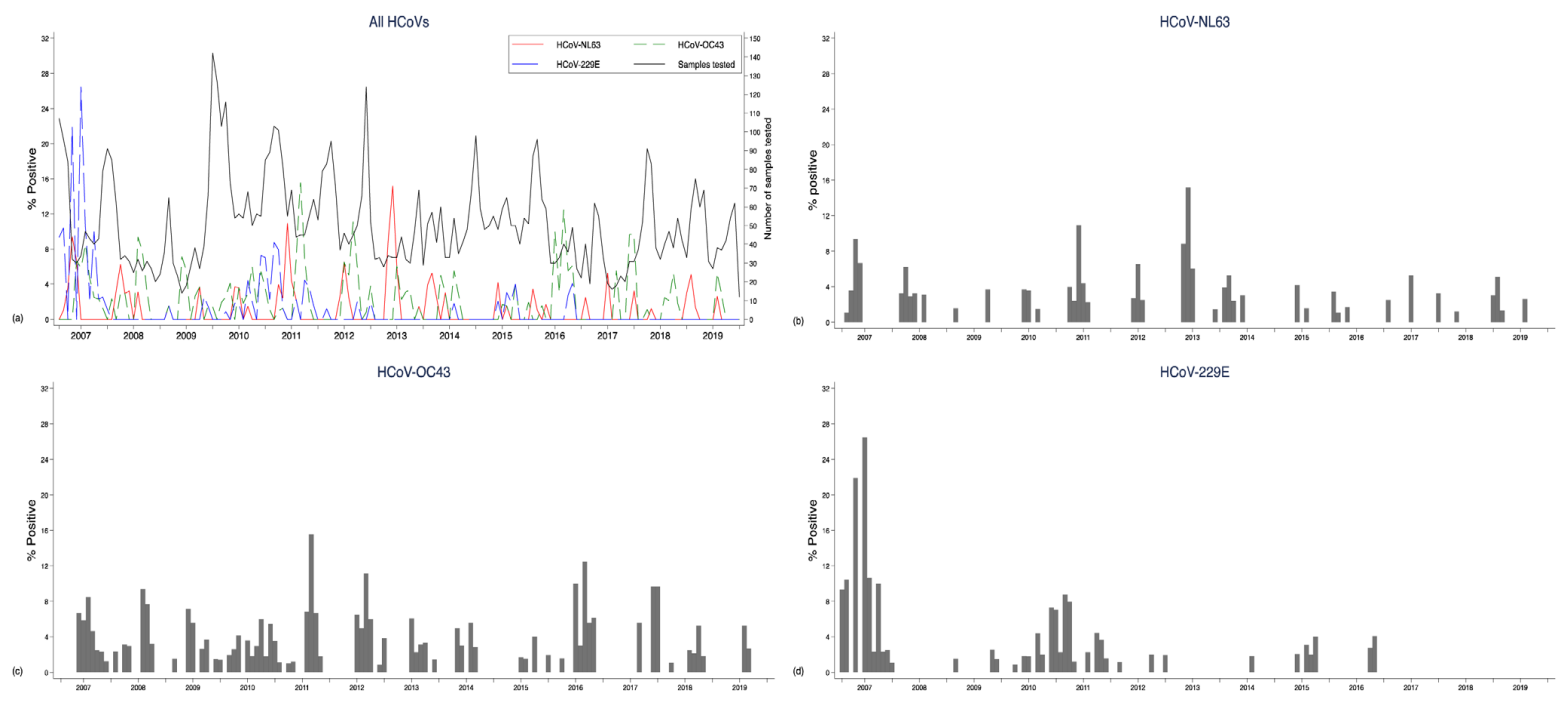
Monthly prevalence (%) pneumonia admissions at Kilifi County Hospital, Kilifi, Kenya 2007–2019 by HCoV type. The panel shows proportions for all HCoVs ( **a**), NL63 ( **b**), OC43 ( **c**) and 229E ( **d**).

**Figure 4.**
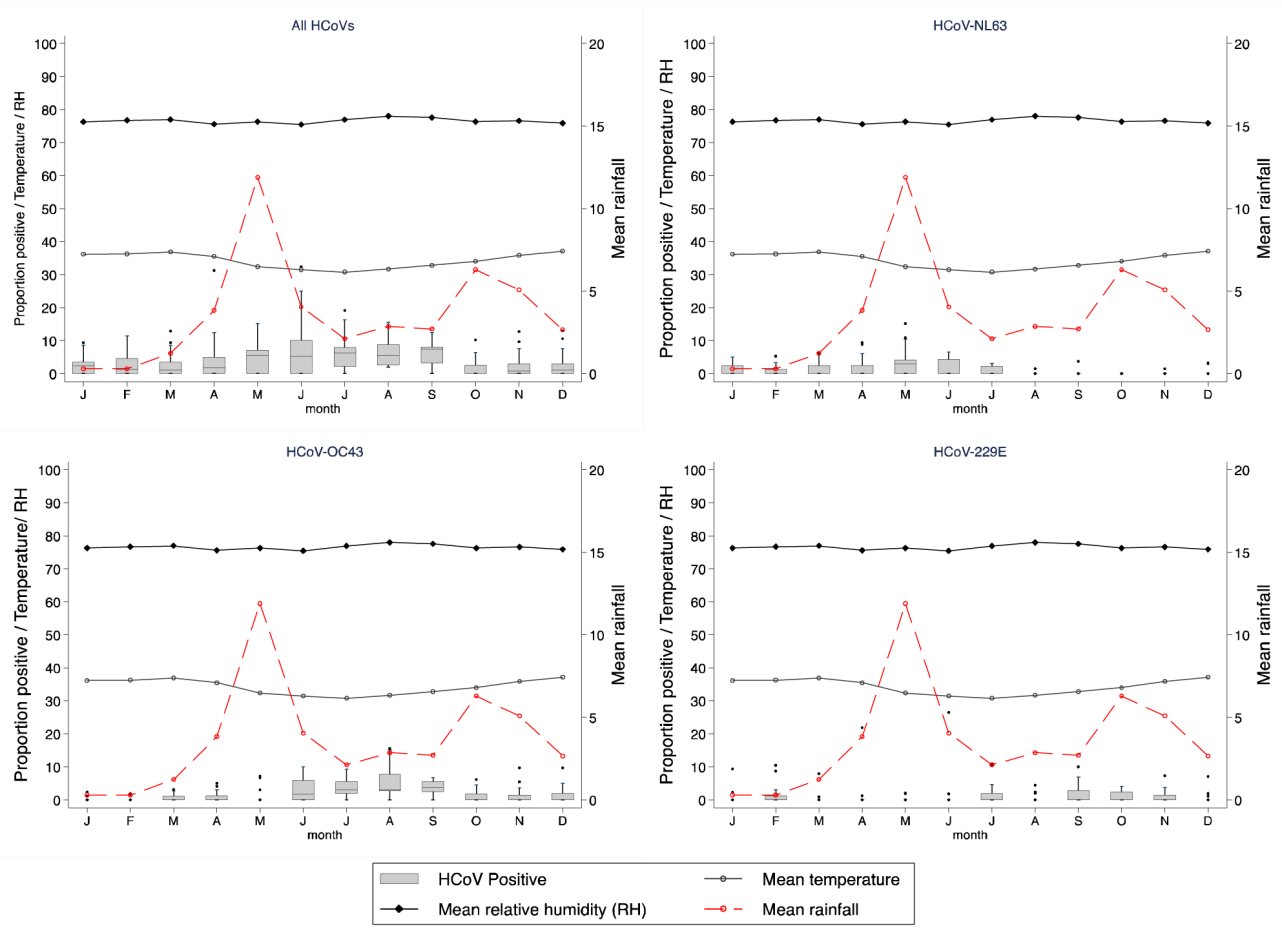
Proportion of monthly positive cases observed at Kilifi County Hospital, Kilifi, Kenya by HCoV type over a period of 13 years (2007–2019). The primary y-axis denotes the proportion of samples positive for HCoV the average monthly maximum temperature in °C and relative humidity (%) while the secondary y-axis denotes the average monthly rainfall in millimetres.

**Figure 5.**
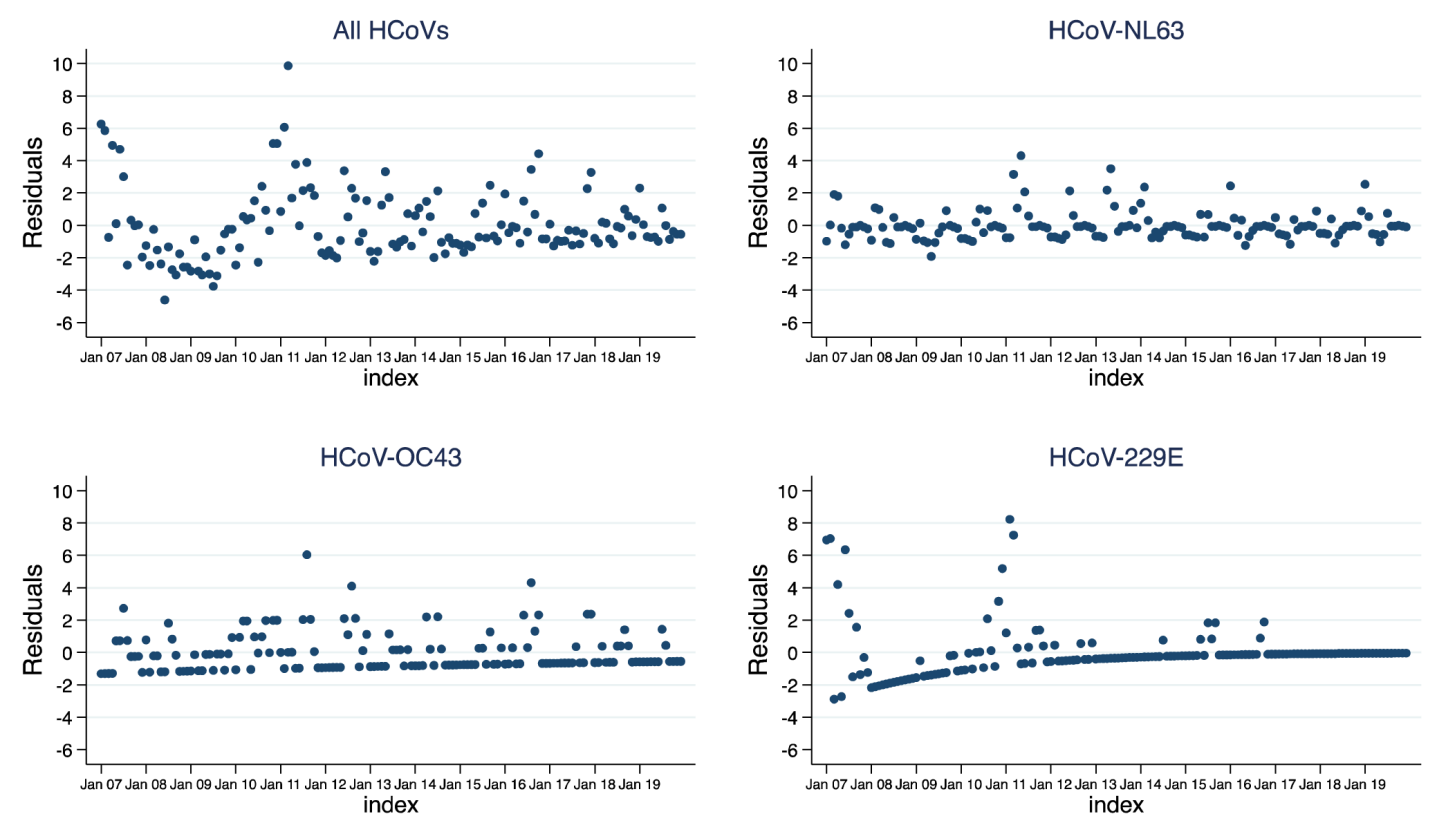
Plot of time series model residuals against month separated by HCoV type using pneumonia surveillance data at Kilifi County Hospital, Kilifi, Kenya 2007–2019. See *Methods* for statistical details.

NL63 and OC43 were observed fairly consistently throughout the surveillance period while fewer cases of HCoV-229E were observed from the middle of 2011 and were not detected subsequent to 2016 ( Figure 3). The highest numbers of cases were observed in the periods April to June for NL63, June to September for OC43 and January to March for 229E. Pooling data for all HCoVs, there were more cases in the colder months (May to September) than the hotter months (October to April) ( Figure 4), as for OC43, but NL63 was more common in the first half of the year, and 229E in the second half of the year. However, time series models did not indicate a seasonal pattern for any of the HCoVs ( Figure 5) over the years. The proportion of samples tested for HCoV did not change over time among those with severe pneumonia ( $\chi_{\left(1\right)}^{2}=3.11;$ p-value = 0.078) but changed among those with very severe pneumonia ( $\chi_{\left(1\right)}^{2}=149.11;$ p-value < 0.001).

De-identified raw data for this study are available as *Underlying data* ^18^.

## Discussion

We have described the circulation patterns of three endemic HCoVs (NL63, OC43 and 229E) in a long-term surveillance study of childhood pneumonia hospitalisations in coastal Kenya. We observed a small proportion of pneumonia admissions positive for one or more HCoVs (3.9%). While 65% of HCoV infections occurred in children in their first year of life (either cumulatively for all HCoVs or for each individual HCoV type), this reflected the age-distribution of pneumonia admissions to the ward. Hence, contrary to other reports ^19^, HCoV did not differ by age and this might reflect no variation in age-specific community incidence of infection in children under five, together with lack of variation in disease severity (likelihood of hospitalisation) across ages, or (ii) variation in age-specific community incidence by age but with a disproportionate probability of hospitalisation. Our reported prevalence is equivalent to that from a long-term hospital surveillance of seasonal coronaviruses in Scotland (4%) ^8^ but lower compared to a multi-centre study across Kenya for the same three HCoVs (26/417 6.2% versus 314/7643 3.9%; $\chi_{\left(1\right)}^{2}=4.43,$ p-value=0.035) ^7^. This latter study included locations with a wide range of climate conditions that might influence prevalence; however, the study was not big enough to stratify by location.

We did not observe seasonal variation of HCoVs compared to some other respiratory viral pathogens like RSV, as previously reported from our site ^14^. In addition, neither peak months for pneumonia admissions nor the long rainy periods (April to July) in Kilifi were associated with HCoV peaks. This is in contrast to data from temperate settings where seasonality of HCoVs has been reported ^8, 20– 22^, with increased occurrence during the colder winter months. The HCoVs we have studied are known to continuously circulate among humans ^1^, although in Kilifi we observed low numbers for all types. Of interest is that pneumonia associated with 229E admissions was not detected in the later years of the surveillance. We attempted to investigate if this was due to primers or probe mismatches. For all the three tested endemic CoVs we did not observe significant mismatches on the primer/probe pairs against data available from GenBank database although this investigation suffered a limitation of few sequences available globally in recent years (2015–2019) and none from East Africa. With the highest numbers and consistent presence compared to NL63 and 229E, our results suggest that OC43 is the predominant HCoV type in the coastal region of Kenya.

The present study did not have a control group by which to assess an aetiological association between the HCoVs and pneumonia. In the PERCH multi-country case-control study ^17^ HCoVs contributed less than 1% of the etiological fraction. In our study the contribution to disease is not known (except for the relatively small set of samples from 2011–13 that were part of the PERCH study), but it is of note that around 50% co-occurred with another respiratory virus (most commonly with RSV), the risk was not age-dependent, there was no clear association between any of the viruses with the any of the specific clinical signs or outcomes investigated and in 26% of deaths with a HCoV detected there was a likely alternative diagnosis to pneumonia. This suggests the presence of incidental HCoV in Kilifi and other clinical groups (hence an under-estimation in this study), though causality of death outcomes was not differentially identified in this study. While we have been able to sequence the virus from a proportion of the positive specimens ^23, 24^, we cannot assume 100% specificity, and even a modest level of false positivity could account for many of the positive diagnoses and argues for caution in interpreting the prevalence estimates. Of relevance also is that few (~1%) of the 314 children positive for at least one HCoV were subsequently HCoV-positive readmissions. While this is a crude analysis which ignores censoring at the start and end of the surveillance, and alternative hospitals where patients may have been admitted, it might be an indicator of low probability of severe reinfection. Alternatively, these readmissions might be children with a prolonged infection having not fully recovered at their first discharge. In addition, HKU1 was only tested during the 24 month period that PERCH study was conducted hence samples tested for HKU1 were considered unrepresentative of the entire study period and therefore not included in this analysis.

Over the surveillance period, we have changed our sample collection and testing methods. This is a limitation; we did not conduct a sensitivity analysis to compare the different PCR methods, and the addition of an OP swab increases the number of viruses found by NP alone (by 14% for HCoVs) ^15^. Some of the observed patterns may have been influenced by these changes. It should be noted that fever, either on history or as measured at the time of admission, was not an inclusion criterion for eligibility, which might have influenced the prevalence of HCoV. If the endemic COVs are strongly associated with symptoms of fever, as is SARS CoV-2, then we might be under-estimating the prevalence due to a selection bias. However, interestingly, only 58% of the HCoV positive cases had axillary temperature of >=37.5°C. A further limitation is that only a fraction (70%) of pneumonia cases was tested for HCoV. We have previously shown that those untested tend to be more severely ill and less likely to be virus positive ^14^. The proportion tested has not substantially changed over time among individuals with severe pneumonia but changed among those with very severe pneumonia. Similarly, we did not observe a difference in the ages of those tested and those untested for HCoV across time. We employed various collection methods and these are known to differ in the range of viruses detected. For example, the addition of an OP swab to an NP swab has been found to increase virus yield ^15^

In conclusion, in this tropical setting we find the three endemic coronaviruses were observe at low prevalence, not dissimilar to influenza and metapneumovirus, but considerably lower that for RSV and human rhinovirus. There was no clear seasonal variation. As the pandemic of COVID-19 takes its course, it is of interest to speculate whether the SARS-CoV-2 virus will become endemic and continuously co-circulate in the human population with the existing HCoVs ^7, 25^. The epidemiology of endemic HCoVs can be used to inform our expectations of SARS-CoV-2 in childhood, its potential severity and inter-species interactions and competition.

## Data availability

### Underlying data

Harvard Dataverse: Replication Data for: Surveillance of endemic human coronaviruses (HCoV-NL63, OC43 and 229E) associated with pneumonia in Kilifi, Kenya. https://doi.org/10.7910/DVN/ZQ1DJY ^18^.

This project contains the following underlying data:

- KCH_paed_ARI_surv_pneumo (CSV). (De-identified underlying data for each patient in the study.)
- KCH_paed_ARI_surv_pneumo-1 (SAV). (De-identified underlying data for each patient in the study.)
- GPOtieno_HCOV_Codebook (PDF). (Data dictionary and codebook.)

The data have been de-identified, and hence lack personally identifiable information. To request access to additional variables from this dataset go to ‘Data Governance’ on http://kemri-wellcome.org/about-us/#ChildVerticalTab15 and submit an ‘External Request’ to the Data Governance Committee ( dgc@kemri-wellcome.org).

### Extended data

Harvard Dataverse: Replication Data for: Surveillance of endemic human coronaviruses (HCoV-NL63, OC43 and 229E) associated with pneumonia in Kilifi, Kenya. https://doi.org/10.7910/DVN/ZQ1DJY ^18^.

This project contains the following extended data:

- 1_descriptive_analysis (DO). (Scripts used to generate information in the tables, data on frequencies, HCOVs virus distributions and proportions.)
- 2_graph_outputs (DO). (Code used to generate charts in the paper.)
- 3_ORs (DO). (Code to fit univariable logistic regression models for each HCoV type.)
- 4_seasonality (DO). (scripts for analyses of HCoV type seasonality.)

Data are available under the terms of the Creative Commons Attribution 4.0 International license (CC-BY 4.0).

## References

[1] van der Hoek L Pyrc K Jebbink MF: Identification of a new human coronavirus. *Nat Med.* 2004;10(4):368–73. 10.1038/nm1024 15034574 PMC7095789

[2] Woo PCY Lau SKP Chu CM: Characterization and Complete Genome Sequence of a Novel Coronavirus, Coronavirus HKU1, from Patients with Pneumonia. *J Virol.* 2005;79(2):884–95. 10.1128/JVI.79.2.884-895.2005 15613317 PMC538593

[3] Tyrrell DA Bynoe ML: Cultivation of a Novel Type of Common-cold Virus in Organ Cultures. *Br Med J.* 1965;1(5448):1467–70. 10.1136/bmj.1.5448.1467 14288084 PMC2166670

[4] Hamre D Procknow JJ: A new virus isolated from the human respiratory tract. *Proc Soc Exp Biol Med.* 1966;121(1):190–3. 10.3181/00379727-121-30734 4285768

[5] Nyiro JU Munywoki P Kamau E: Surveillance of respiratory viruses in the outpatient setting in rural coastal Kenya: baseline epidemiological observations [version 1; peer review: 2 approved]. *Wellcome Open Res.* 2018;3:89. 10.12688/wellcomeopenres.14662.1 30175247 PMC6081997

[6] Berkley JA Munywoki P Ngama M: Viral etiology of severe pneumonia among Kenyan young infants and children. *JAMA.* 2010;303(20):2051–7. 10.1001/jama.2010.675 20501927 PMC2968755

[7] Sipulwa LA Ongus JR Coldren RL: Molecular characterization of human coronaviruses and their circulation dynamics in Kenya, 2009-2012. *Virol J.* 2016;13(1):18. 10.1186/s12985-016-0474-x 26833249 PMC4736488

[8] Nickbakhsh S Ho A Marques DFP: Epidemiology of seasonal coronaviruses: Establishing the context for COVID-19 emergence. *medRxiv.* 2020 10.1101/2020.03.18.20037101 PMC7184404 32296837

[9] Peiris JSM Lai ST Poon LLM: Coronavirus as a possible cause of severe acute respiratory syndrome. *Lancet.* 2003;361(9366):1319–25. 10.1016/s0140-6736(03)13077-2 12711465 PMC7112372

[10] Raj VS Osterhaus ADME Fouchier RAM: MERS: emergence of a novel human coronavirus. *Curr Opin Virol.* 2014;5:58–62. 10.1016/j.coviro.2014.01.010 24584035 PMC4028407

[11] World Health Organization: Coronavirus. [cited 2020 Mar 20]. Reference Source

[12] Fehr AR Perlman S: Coronaviruses: An Overview of Their Replication and Pathogenesis. *Methods Mol Biol.* 2015;1282:1–23. 10.1007/978-1-4939-2438-7_1 25720466 PMC4369385

[13] Scott JAG Bauni E Moisi JC: Profile: The Kilifi Health and Demographic Surveillance System (KHDSS). *Int J Epidemiol.* 2012;41(3):650–7. 10.1093/ije/dys062 22544844 PMC3396317

[14] Nokes DJ Ngama M Bett A: Incidence and Severity of Respiratory Syncytial Virus Pneumonia in Rural Kenyan Children Identified through Hospital Surveillance. *Clin Infect Dis.* 2009;49(9):1341–9. 10.1086/606055 19788358 PMC2762474

[15] Hammitt LL Kazungu S Welch S: Added Value of an Oropharyngeal Swab in Detection of Viruses in Children Hospitalized with Lower Respiratory Tract Infection. *J Clin Microbiol.* 2011;49(6):2318–20. 10.1128/JCM.02605-10 21490188 PMC3122752

[16] Kamau E Agoti CN Lewa CS: Recent sequence variation in probe binding site affected detection of respiratory syncytial virus group B by real-time RT-PCR. *J Clin Virol.* 2017;88:21–5. 10.1016/j.jcv.2016.12.011 28107671 PMC5331890

[17] Pneumonia Etiology Research for Child Health (PERCH) Study Group: Causes of severe pneumonia requiring hospital admission in children without HIV infection from Africa and Asia: the PERCH multi-country case-control study. *Lancet.* 2019;394(10200):757–79. 10.1016/S0140-6736(19)30721-4 31257127 PMC6727070

[18] Otieno GP Murunga N Agoti C: Replication Data for: Surveillance of endemic human coronaviruses (HCoV-NL63, OC43 and 229E) associated with pneumonia in Kilifi, Kenya.Harvard Dataverse.2020 10.7910/DVN/ZQ1DJY PMC7512035 32995556

[19] Ogimi C Englund JA Bradford MC: Characteristics and Outcomes of Coronavirus Infection in Children: The Role of Viral Factors and an Immunocompromised State. *J Pediatr Infect Dis Soc.* 2019;8(1):21–8. 10.1093/jpids/pix093 29447395 PMC6437838

[20] Gaunt ER Hardie A Claas ECJ: Epidemiology and Clinical Presentations of the Four Human Coronaviruses 229E, HKU1, NL63, and OC43 Detected over 3 Years Using a Novel Multiplex Real-Time PCR Method. *J Clin Microbiol.* 2010;48(8):2940–7. 10.1128/JCM.00636-10 20554810 PMC2916580

[21] Hoek LVD Pyrc K Berkhout B: Human coronavirus NL63, a new respiratory virus. *FEMS Microbiol Rev.* 2006;30(5):760–73. 10.1111/j.1574-6976.2006.00032.x 16911043 PMC7109777

[22] Aldridge RW Lewer D Beale S: Seasonality and immunity to laboratory-confirmed seasonal coronaviruses (HCoV-NL63, HCoV-OC43, and HCoV-229E): results from the Flu Watch cohort study [version 1; peer review: 2 approved with reservations]. *Wellcome Open Res.* 2020;5:52 10.12688/wellcomeopenres.15812.1 PMC7786426 33447664

[23] Kamau E Luka MM Laurent ZR de: Genome Sequences of Human Coronavirus OC43 and NL63, Associated with Respiratory Infections in Kilifi, Kenya. *Microbiol Resour Announc.* 2019;8(46):e00730-19. 10.1128/MRA.00730-19 31727697 PMC6856263

[24] Kiyuka PK Agoti CN Munywoki PK: Human Coronavirus NL63 Molecular Epidemiology and Evolutionary Patterns in Rural Coastal Kenya. *J Infect Dis.* 2018;217(11):1728–39. 10.1093/infdis/jiy098 29741740 PMC6037089

[25] Killerby ME Biggs HM Haynes A: Human coronavirus circulation in the United States 2014-2017. *J Clin Virol.* 2018;101:52–6. 10.1016/j.jcv.2018.01.019 29427907 PMC7106380

