# Supplementary material for: Surveillance of endemic human coronaviruses (HCoV-NL63, OC43 and 229E) associated with childhood pneumonia in Kilifi, Kenya
Source: Wellcome Open Res. 2020 Sep 22;5:150. Originally published 2020 Jun 26. [Version 2] doi: 10.12688/wellcomeopenres.16037.2 (PMC7512035; doi:10.12688/wellcomeopenres.16037.2)
Supplement: Peer review history

# Peer review: Reviewer response for version 2

**DOI:** 10.21956/wellcomeopenres.17921.r40553

**Published:** 2020-09-23

**Authors:** David P. Moore

**Affiliations:** 1 Medical Research Council, Respiratory and Meningeal Pathogens Research Unit, University of the Witwatersrand, Johannesburg, South Africa; 2 Department of Paediatrics and Child Health, Chris Hani Baragwanath Academic Hospital, University of the Witwatersrand, Johannesburg, South Africa

Thank you for the opportunity of reviewing the authors' revised manuscript. This is a valuable addition to the existing literature on the burden of hospitalised pneumonia in children <5 years of age that is associated with the three endemic human coronaviruses studied.

Is the work clearly and accurately presented and does it cite the current literature?

Yes

If applicable, is the statistical analysis and its interpretation appropriate?

Yes

Are all the source data underlying the results available to ensure full reproducibility?

Yes

Is the study design appropriate and is the work technically sound?

Yes

Are the conclusions drawn adequately supported by the results?

Partly

Are sufficient details of methods and analysis provided to allow replication by others?

Yes

Reviewer Expertise:

Paediatric pneumonia aetiology; paediatric infectious diseases.

I confirm that I have read this submission and believe that I have an appropriate level of expertise to confirm that it is of an acceptable scientific standard.

# Peer review: Reviewer response for version 2

**DOI:** 10.21956/wellcomeopenres.17921.r40552

**Published:** 2020-09-23

**Authors:** Sema Nickbakhsh

**Affiliations:** 1 MRC-University of Glasgow Centre for Virus Research, Institute of Infection, Immunity and Inflammation, College of Medical, Veterinary and Life Sciences, University of Glasgow, Glasgow, UK

I am satisfied that the authors have sufficiently addressed all the concerns raised.

 One minor comment: reference #8 is available in peer reviewed published form if the authors wish to update their reference list: https://doi.org/10.1093/infdis/jiaa185 ^1^.

Is the work clearly and accurately presented and does it cite the current literature?

Yes

If applicable, is the statistical analysis and its interpretation appropriate?

Yes

Are all the source data underlying the results available to ensure full reproducibility?

Yes

Is the study design appropriate and is the work technically sound?

Yes

Are the conclusions drawn adequately supported by the results?

Partly

Are sufficient details of methods and analysis provided to allow replication by others?

Partly

Reviewer Expertise:

Temporal and spatial dynamics of infectious diseases; epidemiology of viral respiratory infections

I confirm that I have read this submission and believe that I have an appropriate level of expertise to confirm that it is of an acceptable scientific standard.

## References

[1] : Epidemiology of Seasonal Coronaviruses: Establishing the Context for the Emergence of Coronavirus Disease 2019. *The Journal of Infectious Diseases*.2020;222(1) : 10.1093/infdis/jiaa185 17-25 10.1093/infdis/jiaa185 PMC718440432296837

# Peer review: Reviewer response for version 1

**DOI:** 10.21956/wellcomeopenres.17592.r39427

**Published:** 2020-07-21

**Authors:** Sema Nickbakhsh

**Affiliations:** 1 MRC-University of Glasgow Centre for Virus Research, Institute of Infection, Immunity and Inflammation, College of Medical, Veterinary and Life Sciences, University of Glasgow, Glasgow, UK

This study describes patterns of endemic human coronavirus detections over a thirteen year period among paediatric cases of pneumonia in coastal Kenya.

 The authors should be commended for the impressive collation of clinical features, discharge diagnosis, and hospitalisation outcomes alongside multiplex PCR tests which is rarely available for a study spanning this length of time.

 The manuscript is well written and clearly presented throughout. I do however have some concerns requiring clarification.

1. The authors mention in Methods that a variety of specimen types were collected. Could the authors clarify whether this is at the individual patient level? If relevant, I suggest authors describe how duplication of samples from individual patients was handled in the analyses (since this can affect probabilty of virus detection and violates statistical assumptions of independent data points).
2. Suggest authors add to Methods a brief outline of the changes in testing procedure over time (as mentioned in the Discussion) to aid interpretation of detection trends, as different specimens are likely associated with different probabilities of virus detection.
3. It is unclear why the laboratory screen omitted HCoV-HKU1. Suggest the authors include a comment on the anticipated influence on the overall detection of HCoV.
4. It is unclear whether exclusion of untested discharged patients (presumably milder cases) have led to an under-estimation of HCoV detection proportions. Suggest authors add a statement to describe any anticipated influence of this exclusion.
5. The % of pneumonia cases with HCoV detected is clearly low. However, it would be helpful if the authors could discuss this result in the context of other common respiratory viruses detected among cases of pneumonia in this setting, such as influenza and RSV.
6. Discussion page 6: “...suggests age is not a risk factor for coronavirus associated pneumonia hospital admission”. The role of age as a risk factor for pneumonia cannot be determined here without a non-pneumonia control group. Suggest authors reword this statement and consider whether the lack of variation in age-specific proportions may potentially reflect (i) no variation in age-specific community incidence of infection in children under five, together with lack of variation in severity (likelihood of hospitalisation) across ages, or (ii) variation in age-specific community incidence by age but with a disproportionate probability of hospitalisation.
7. Discussion page 9: “...and in 26% of deaths with a HCoV detected there was a likely alternative diagnosis to pneumonia”. This seems to suggest the role of HCoV on overall hospital burden may be under-estimated by pneumonia cases. Although not an aim of the study, I suggest the authors consider adding a brief comment regarding the generalisability of their findings to other clinical groups to place the results into a wider context.
8. Discussion page 9: Can the authors comment on the possibility that readmissions reflect prolonged virus shedding, rather than indicating reinfection? Longest duration between admissions of 21 days possibly bordering an anticipated peak detection within first few weeks (e.g. for SARS-CoV-2 ^1^). Suggest authors consider adding a brief statement to include this possibility.
9. Discussion page 10: “ fever... was not an inclusion criterion for eligibility, which might have influenced the prevalence of HCoV”. Suggest authors make clear the anticipated direction of effect on the prevalence - presumably underestimation?
10. Discussion page 10: “...there was a significant age difference for those tested and those untested for HCoV across time.” This statement seems to be at odds with Results page 3 “..cases untested did not differ from those tested in age distribution..” Suggest the authors reword as appropriate.
11. Conclusions page 10: “...little evidence of a substantial aetiological contribution...”: The aetiological role of HCoV cannot be established without a control group (as the authors do mention on page 9) i.e. it is not known whether the prevalence of HCoV is lower among cases of ARI without pneumonia. Suggest authors rephrase to put the low HCoV detections within the context of relative contribution of pathogens/other causes to disease aetiology i.e. most pneumonia cases are attributable to other causes.
12. Although presumably outside the scope of this study to analyse, I suggest the authors consider commenting briefly on any potential role of coinfecting respiratory bacteria on HCoV-associated pneumonia in this population; bacterial coinfection may influence the chance of pneumonia/testing for viruses.

Is the work clearly and accurately presented and does it cite the current literature?

Yes

If applicable, is the statistical analysis and its interpretation appropriate?

Yes

Are all the source data underlying the results available to ensure full reproducibility?

Yes

Is the study design appropriate and is the work technically sound?

Yes

Are the conclusions drawn adequately supported by the results?

Partly

Are sufficient details of methods and analysis provided to allow replication by others?

Partly

Reviewer Expertise:

Temporal and spatial dynamics of infectious diseases; epidemiology of viral respiratory infections

I confirm that I have read this submission and believe that I have an appropriate level of expertise to confirm that it is of an acceptable scientific standard, however I have significant reservations, as outlined above.

## References

[1] : Interpreting Diagnostic Tests for SARS-CoV-2. *JAMA*.2020; 10.1001/jama.2020.8259 10.1001/jama.2020.8259 32374370

# Response

**Published:** 2020-09-07

**Authors:** Grieven Otieno

**Affiliations:** KEMRI-Wellcome Trust, Kenya

We wish to thank the reviewer for the detailed reviewer of the manuscript and the suggested changes/edits. Please see below our responses:

  

1. The authors mention in Methods that a variety of specimen types were collected. Could the authors clarify whether this is at the individual patient level? If relevant, I suggest authors describe how duplication of samples from individual patients was handled in the analyses (since this can affect probabilty of virus detection and violates statistical assumptions of independent data points).

 

**Response**: We collected one sample type per person, but the type varied over time, and we have indicated this in the Methods text of the manuscript

  

1. Suggest authors add to Methods a brief outline of the changes in testing procedure over time (as mentioned in the Discussion) to aid interpretation of detection trends, as different specimens are likely associated with different probabilities of virus detection.

 

**Response** *: We have made the change to the methods section as suggested by the reviewer.*

  

1. It is unclear why the laboratory screen omitted HCoV-HKU1. Suggest the authors include a comment on the anticipated influence on the overall detection of HCoV.

 

**Response** *: We added a comment to the limitations of the study.*

  

1. It is unclear whether exclusion of untested discharged patients (presumably milder cases) have led to an under-estimation of HCoV detection proportions. Suggest authors add a statement to describe any anticipated influence of this exclusion.

 

**Response**: We recognise that eligible but untested individuals represent a possible bias. We state that the untested tended to be the more severe, and these are an important group, whose omission might lead to an under-estimate of the the role of HCoVs in sever disease. Note however, that the prevalence of HCoV infection (any type) was less prevalent amongst those with hypoxemia (a feature of very severe pneumonia).

  

1. The % of pneumonia cases with HCoV detected is clearly low. However, it would be helpful if

the authors could discuss this result in the context of other common respiratory viruses

 detected among cases of pneumonia in this setting, such as influenza and RSV.

**Response**: We have previously presented in several papers that relative to RSV and rhinovirus the prevalence of all other viruses (including influenza) is low and not dissimilar to that of the HCoVs - Berkley et al JAMA 2010 May 26;303(20):2051-7.  doi: 10.1001/jama.2010.675 and Onyango et al  J Infect Dis . 2012 Dec 15;206 Suppl 1(Suppl 1):S61-7. doi: 10.1093/infdis/jis536,  and  Otieno et al BMC Infect Dis  . 2019 Aug 30;19(1): 757.doi: 10.1186/s12879-019-4381-9

  

1. Discussion page 6: “...suggests age is not a risk factor for coronavirus associated pneumonia

hospital admission”. The role of age as a risk factor for pneumonia cannot be determined here without a non-pneumonia control group. Suggest authors reword this statement and consider whether the lack of variation in age-specific proportions may potentially reflect (i) no variation in age-specific community incidence of infection in children under five, together with lack of variation in severity (likelihood of hospitalisation) across ages, or (ii) variation in age-specific community incidence by age but with a disproportionate probability of

 hospitalisation.

**Response** *: We have made the change as suggested by the reviewer.*

  

1. Discussion page 9: “...and in 26% of deaths with a HCoV detected there was a likely alternative diagnosis to pneumonia”. This seems to suggest the role of HCoV on overall hospital burden may be under-estimated by pneumonia cases. Although not an aim of the study, I suggest the authors consider adding a brief comment regarding the generalisability of their findings to other clinical groups to place the results into a wider context.

 

**Response** *: We have made the change as suggested by the reviewer.*

  

1. Discussion page 9: Can the authors comment on the possibility that readmissions reflect

prolonged virus shedding, rather than indicating reinfection? Longest duration between

 admissions of 21 days possibly bordering an anticipated peak detection within first few

 weeks (e.g. for SARS-CoV-21). Suggest authors consider adding a brief statement to include

 this possibility.

**Response**: It is possible these are prolonged infections rather than reinfections and we have added the following to the text in the discussion: "...probability of severe reinfection. Alternatively, these readmissions might be children with a prolonged infection having not fully recovered at their  first discharge"

  

1. Discussion page 10: “ fever... was not an inclusion criterion for eligibility, which might have

influenced the prevalence of HCoV”. Suggest authors make clear the anticipated direction of

 effect on the prevalence - presumably underestimation?

**Response**: This is possible, but we don't know that these endemic HCoVs behave like SRAS-CoV-2 ie are strongly associated with fever, hence again difficult to speculate as to which direction. We therefore added this statement to the limitations “ If the endemic COVs are strongly associated with symptoms of fever, as is SARS=CoV-2, then we might be under-estimating the prevalence due to a selection bias”.

 Discussion page 10: “...there was a significant age difference for those tested and those untested for HCoV across time.” This statement seems to be at odds with Results page 3 “..cases untested did not differ from those tested in age distribution..” Suggest the authors reword as appropriate.

**Response** *: We have made the change as suggested by the reviewer.*

 Conclusions page 10: “...little evidence of a substantial aetiological contribution...”: The aetiological role of HCoV cannot be established without a control group (as the authors do mention on page 9) i.e. it is not known whether the prevalence of HCoV is lower among cases of ARI without pneumonia. Suggest authors rephrase to put the low HCoV detections within the context of relative contribution of pathogens/other causes to disease aetiology i.e. most pneumonia cases are attributable to other causes.

**Response** *: We have made the change as suggested by the reviewer.*

 Although presumably outside the scope of this study to analyse, I suggest the authors consider commenting briefly on any potential role of coinfecting respiratory bacteria on HCoV-associated pneumonia in this population; bacterial coinfection may influence the  chance of pneumonia/testing for viruses.

**Response**: We had very low proportions of bacterial infections among the positive cases: NL63 (2/74=2.7%), OC43 (3/129=2.3%), 229E (2/99=2.02%). We feel there is too little co-infection to allow speculation on this.

# Peer review: Reviewer response for version 1

**DOI:** 10.21956/wellcomeopenres.17592.r39270

**Published:** 2020-07-06

**Authors:** David P. Moore

**Affiliations:** 1 Medical Research Council, Respiratory and Meningeal Pathogens Research Unit, University of the Witwatersrand, Johannesburg, South Africa; 2 Department of Paediatrics and Child Health, Chris Hani Baragwanath Academic Hospital, University of the Witwatersrand, Johannesburg, South Africa

Thank you for the opportunity of reviewing this well-written manuscript by the accomplished research team at Kilifi. They describe the prevalence of endemic human coronavirus (HCoV) pneumonia hospitalisations in children <5 years of age in Kilifi over a 13-year surveillance period.

**Major comments:**

1. Why was HCoV-HKU1 not included in the surveillance of endemic human coronaviruses in this study? Lack of testing for HCoV-HKU1 should be mentioned as a limitation of the study.
  - PERCH samples were included in the analysis, which would have included HCoV-HCoV-HKU1: mention should be made that, as HKU1 was only tested for in the 24-month PERCH surveillance period, it was considered to be under-represented over the study time period and was therefore not included in the analysis.
2. Mention is made of a variety of specimen types that were tested for respiratory viruses in the study. Is there evidence to suggest that any one type gives better yields than others? This should be elaborated on briefly in the discussion section, potentially as a limitation of the study.
3. The authors mention that an alternative attribution could be made in 26% of cases that died with a positive detection of HCoV in their study (page 9, left hand column, third sentence); however, this in not explored at all in the Results Section. This should be presented in the Results section, so as to contextualise the assertion made in the Discussion.

**Minor comments:**

1. Suggest change the title to reflect that paediatric pneumonia was the focus of attention in this study. Suggest “Surveillance of endemic human coronaviruses (HCoV-NL63, OC43 and 229E) associated with childhood pneumonia in Kilifi, Kenya”.
2. Correct the redundant double comma in affiliation 2 at the top of the title page of the manuscript.
3. In the results section of the Abstract, qualify that 3.9% of the pneumonia admissions tested positive for at least one of the three endemic HCoVs surveyed in the study.
4. It is unclear what is meant by “no age-dependence in the proportion testing positive” for HCoV in the abstract. Suggest omit this ambiguous phrase. Rather state “… n=208), and there was no clear seasonal pattern of HCoV infection. HCoV-OC43 was predominant…”
5. Suggest reword the Abstract conclusions as “… co-occurring with another respiratory virus, no clear seasonal pattern, and with the age-distribution…”
6. Suggest omit the speculation as to the impact of SARS-CoV-2 at the end of the last sentence of the Abstract.
7. At the end of the Introduction, suggest reword as “Our study aims to describe the circulation patterns of three of the endemic HCoVs (OC43, 229E, and NL63) over time…”
8. Suggest omit the definite article in the first sentence of the Methods section, i.e. “… in 2007 for long-term continuous respiratory virus surveillance among…”
9. Consider amending slight punctuation errors in page 3, including:
  - Left column, six lines from the bottom: “… ≥37.5°C, oxygen…”
  - Right column, second line: “… alive or dead).”
10. There are two mentions of which viruses were tested for on page 3: suggest concatenate into one mention (probably best placed in the “Laboratory methods” paragraph). Reword at the bottom of page 3 on the right hand column to “… or very severe), laboratory test results, and hospitalisation outcomes…”
11. The link to code in *Extended data* is not currently active in the manuscript: please correct this.
12. At the bottom of page 3, suggest reword as “Untested cases did not differ… more likely to have very severe pneumonia (40.0% versus 28.3%; *Χ* ^2^=152.5, P<0.001).”
  - Why resort to the use of Fisher’s exact P-value in the analysis of tested and untested cases (the Chi-square P-value is every bit as “significant”)?
13. Children are not “samples”: suggest reword the sentence at the top of page 4 as “Of the 7957 children tested, 5312 (66.7%) were aged <1 year, 1454…”
14. Suggest clarify the meaning of the second sentence on page 4 as follows: “The proportion of tested individuals with fever (axillary temperature ≥37.5°C), cough, and difficulty breathing was 58.3%, 83.2% and 92.6%, respectively.”
15. On page 4, suggest reword the third last line on the left hand column as “… testing positive for HCoV (3.9%) did not vary with age group…”
16. In Table 1, suggest present Age (months) as median and interquartile range only.
17. In Table 1, rather use the term “Difficulty breathing” which is more widely used than “Breathing difficulty”, also use “Prostration” rather than “Prostrate/unconscious”.
18. There are typos in the footnote to Table 1: “tde” should be “the”; “breatding difficulty” should be “difficulty breathing”; “witd” should be “with”.
19. Suggest reword the first sentence under the heading “Clinical outcomes of HCoV-infected patients” on page 5 as: “… positive for at least one of the three endemic HCoVs investigated for in this study were admitted to…”
20. In Figure 1, the HCoV-OC43 figure y-axis requires correction of spelling of the word “immunosuppression”; the HCoV-229E figure requires change of the “Malnutrition & immunosuppression” label on the y-axis to have an initial capital “M”.
21. Suggest reword the first sentence under the heading “Temporal patterns of different HCoVs” on page 5 as: “… middle of 2011, and were not detected subsequent to 2016 (Figure 3).”
22. In Table 2, suggest add in an analysis for all HCoVs combined.
23. Suggest reword the first sentence of the Discussion as “… patterns of three endemic HCoVs (NL63, OC43 and 229E) in a… positive for one or more of the HCoVs that were investigated for (3.9%).”
24. Check the calculation of the comparison between Sipulwa et al and the current study, in the second line on the right hand column on page 9: I get *Χ* ^2^=3.92, p=0.048.
25. In the second sentence of the second paragraph on the right column on page 9, suggest reword to “… nor the long rainy periods (April to July) in Kilifi were associated with HCoV peaks.”
26. Suggest reword the fifth sentence in the right hand column on page 9 as “Of interest is that pneumonia associated with 229E was not detected in the later years of surveillance.”
27. Please correct the spelling or “axillary” in the footnote to Table 2.
28. The authors do not consider differences in participant recruitment as being a potential explanation as to why 229E-associated pneumonia was not detected subsequent to 2016: were there systematic differences in participant recruitment strategies that might have impacted on detection of this virus?
29. Suggest reword the third sentence on the right hand column on page 10 as “It should be noted that fever, either on history or as measured at the time of admission, was not an inclusion criterion…”

Is the work clearly and accurately presented and does it cite the current literature?

Yes

If applicable, is the statistical analysis and its interpretation appropriate?

Yes

Are all the source data underlying the results available to ensure full reproducibility?

Yes

Is the study design appropriate and is the work technically sound?

Yes

Are the conclusions drawn adequately supported by the results?

Partly

Are sufficient details of methods and analysis provided to allow replication by others?

Yes

Reviewer Expertise:

Paediatric pneumonia aetiology; paediatric infectious diseases.

I confirm that I have read this submission and believe that I have an appropriate level of expertise to confirm that it is of an acceptable scientific standard, however I have significant reservations, as outlined above.

# Response

**Published:** 2020-09-07

**Authors:** Grieven Otieno

**Affiliations:** KEMRI-Wellcome Trust, Kenya

Major comments:

1. Why was HCoV-HKU1 not included in the surveillance of endemic human coronaviruses in this

study? Lack of testing for HCoV-HKU1 should be mentioned as a limitation of the study. PERCH samples were included in the analysis, which would have included HCoV-HCoV-HKU1: mention should be made that, as HKU1 was only tested for in the 24-month PERCH surveillance period, it was considered to be under-represented over the study time period and was therefore not included in the analysis.

**Response** *: We thank the reviewer for pointing this out. We have edited the manuscript as advised by the reviewer.*

  

1. Mention is made of a variety of specimen types that were tested for respiratory viruses in the study. Is there evidence to suggest that any one type gives better yields than others? This should be elaborated on briefly in the discussion section, potentially as a limitation of the study.

 

**Response** *: We thank the reviewer for pointing this out. We have added the following statement to the limitations of the study*

*“We employed various collection methods and these are known to differ in the range of viruses detected. For example, the addition of an OP swab to an NP  swab has been found to increase virus yield*”

  

1. The authors mention that an alternative attribution could be made in 26% of cases that died with a

positive detection of HCoV in their study (page 9, left hand column, third sentence); however, this

 in not explored at all in the Results Section. This should be presented in the Results section, so as

 to contextualise the assertion made in the Discussion.

**Response**: *We have expanded the statements in the results section to refer to the proportion of individuals and made adjustments to the discussion section.*

 Minor comments:

  

1. Suggest change the title to reflect that paediatric pneumonia was the focus of attention in this

study. Suggest “Surveillance of endemic human coronaviruses (HCoV-NL63, OC43 and 229E)

 associated with childhood pneumonia in Kilifi, Kenya”.

**Response** *: We have made the change as suggested by the reviewer.*

  

1. Correct the redundant double comma in affiliation 2 at the top of the title page of the manuscript.

 

**Response** *: We have made the change as suggested by the reviewer.*

  

1. In the results section of the Abstract, qualify that 3.9% of the pneumonia admissions tested positive

for at least one of the three endemic HCoVs surveyed in the study.

**Response** *: We have made the change as suggested by the reviewer.*

  

1. It is unclear what is meant by “no age-dependence in the proportion testing positive” for HCoV in

the abstract. Suggest omit this ambiguous phrase. Rather state “… n=208), and there was no clear

 seasonal pattern of HCoV infection. HCoV-OC43 was predominant…”

**Response**: We have adjusted the statement in the abstract but would wish to retain the rest. Although there were more cases in the younger age groups this simply reflected the more eligible admissions.

  

1. Suggest reword the Abstract conclusions as “… co-occurring with another respiratory virus, no

clear seasonal pattern, and with the age-distribution…”

**Response** *: We have made the change as suggested by the reviewer.*

  

1. Suggest omit the speculation as to the impact of SARS-CoV-2 at the end of the last sentence of the

Abstract.

**Response** *: We have made the change as suggested by the reviewer.*

  

1. At the end of the Introduction, suggest reword as “Our study aims to describe the circulation

patterns of three of the endemic HCoVs (OC43, 229E, and NL63) over time…”

**Response** *: We have made the change as suggested by the reviewer.*

  

1. Suggest omit the definite article in the first sentence of the Methods section, i.e. “… in 2007 for

long-term continuous respiratory virus surveillance among…”

**Response** *: We have made the change as suggested by the reviewer.*

  

1. Consider amending slight punctuation errors in page 3, including:

Left column, six lines from the bottom: “… ≥37.5°C, oxygen…”

 Right column, second line: “… alive or dead).”

**Response** *: We have made the change as suggested by the reviewer.*

  

1. There are two mentions of which viruses were tested for on page 3: suggest concatenate into one

mention (probably best placed in the “Laboratory methods” paragraph). Reword at the bottom of

 page 3 on the right hand column to “… or very severe), laboratory test results, and hospitalisation

 outcomes…”

**Response** *: We have made the change as suggested by the reviewer.*

  

1. The link to code in Extended data is not currently active in the manuscript: please correct this.

 

**Response**: *We have corrected the error with the  link and it is now active.*

  

1. At the bottom of page 3, suggest reword as “Untested cases did not differ… more likely to have

very severe pneumonia (40.0% versus 28.3%; Χ =152.5, P<0.001).” Why resort to the use of Fisher’s exact P-value in the analysis of tested and untested cases (the Chi-square P-value is every bit as “significant”)?

**Response**: *Fisher’s exact p-value is reported because of the low expected counts (<5)  in the older age bands*

  

1. Children are not “samples”: suggest reword the sentence at the top of page 4 as “Of the 7957

children tested, 5312 (66.7%) were aged <1 year, 1454…”

**Response** *: We have made the change as suggested by the reviewer.*

  

1. Suggest clarify the meaning of the second sentence on page 4 as follows: “The proportion of

tested individuals with fever (axillary temperature ≥37.5°C), cough, and difficulty breathing was

 58.3%, 83.2% and 92.6%, respectively.”

**Response** *: We have made the change as suggested by the reviewer.*

  

1. On page 4, suggest reword the third last line on the left hand column as “… testing positive for

HCoV (3.9%) did not vary with age group…”

**Response** *: We have made the change as suggested by the reviewer.*

  

1. In Table 1, suggest present Age (months) as median and interquartile range only.

 

**Response** *: We have made the change as suggested by the reviewer.*

  

1. In Table 1, rather use the term “Difficulty breathing” which is more widely used than “Breathing

difficulty”, also use “Prostration” rather than “Prostrate/unconscious”.

**Response** *: We have made the change as suggested by the reviewer.*

  

1. There are typos in the footnote to Table 1: “tde” should be “the”; “breatding difficulty” should be

“difficulty breathing”; “witd” should be “with”.

**Response** *: We have made the change as suggested by the reviewer.*

  

1. Suggest reword the first sentence under the heading “Clinical outcomes of HCoV-infected patients”

on page 5 as: “… positive for at least one of the three endemic HCoVs investigated for in this study

 were admitted to…”

**Response** *: We have made the change as suggested by the reviewer.*

  

1. In Figure 1, the HCoV-OC43 figure y-axis requires correction of spelling of the word

“immunosuppression”; the HCoV-229E figure requires change of the “Malnutrition & immunosuppression” label on the y-axis to have an initial capital “M”.

**Response** *: We have made the change as suggested by the reviewer.*

  

1. Suggest reword the first sentence under the heading “Temporal patterns of different HCoVs” on

page 5 as: “… middle of 2011, and were not detected subsequent to 2016 (Figure 3).”

**Response** *: We have made the change as suggested by the reviewer.*

  

1. In Table 2, suggest add in an analysis for all HCoVs combined.

 

**Response** *: We have made the change as suggested by the reviewer.*

  

1. Suggest reword the first sentence of the Discussion as “… patterns of three endemic HCoVs

(NL63, OC43 and 229E) in a… positive for one or more of the HCoVs that were investigated for

 (3.9%).”

**Response** *: We have made the change as suggested by the reviewer.*

  

1. Check the calculation of the comparison between Sipulwa et al and the current study, in the

second line on the right hand column on page 9: I get Χ =3.92, p=0.048.

**Response** *: We have made the change as suggested by the reviewer.*

  

1. In the second sentence of the second paragraph on the right column on page 9, suggest reword to

“… nor the long rainy periods (April to July) in Kilifi were associated with HCoV peaks.”

**Response** *: We have made the change as suggested by the reviewer.*

  

1. Suggest reword the fifth sentence in the right hand column on page 9 as “Of interest is that

pneumonia associated with 229E was not detected in the later years of surveillance.”

**Response** *: We have made the change as suggested by the reviewer.*

  

1. Please correct the spelling or “axillary” in the footnote to Table 2.

**Response** *: We have made the change as suggested by the reviewer.*

  

1. The authors do not consider differences in participant recruitment as being a potential explanation as to why 229E-associated pneumonia was not detected subsequent to 2016: were there systematic differences in participant recruitment strategies that might have impacted on detection of this virus?

**Response** *:  We have made the change as suggested by the reviewer.*

  

1. Suggest reword the third sentence on the right hand column on page 10 as “It should be noted that fever, either on history or as measured at the time of admission, was not an inclusion criterion…”

 

**Response** *:  We have made the change as suggested by the reviewer.*
